# Supplementary material for: Collaborative intelligence in AI: Evaluating the performance of a council of AIs on the USMLE
Source: PLOS Digit Health. 2025 Oct 9;4(10):e0000787. doi: 10.1371/journal.pdig.0000787 (PMC12510544; doi:10.1371/journal.pdig.0000787)
Supplement: S2 File — (ZIP) [file pdig.0000787.s002.zip › S2 Data Raw data files containing unprocessed question inputs and LLM outputs including transcripts of deliberations/STEP3_Question-39-1_Type-MC-NJ.docx]

**Facilitator** **[Round 1]:** A 70-year-old woman comes to the office because of a 1-month history of gradually worsening shortness of breath. Medical history is significant for hypertension and hyperlipidemia. Routine medications are atorvastatin and lisinopril. The patient appears to be in respiratory distress. Vital signs are temperature 36.7°C (98.0°F), pulse 122/min and irregularly irregular, respirations 28/min, and blood pressure 144/88 mm Hg. Pulse oximetry on room air shows an oxygen saturation of 90%. Pulmonary examination discloses dullness to percussion three-quarters of the way up on the left. Results of laboratory studies are shown: Serum Blood Urea nitrogen 29 mg/dL Hematocrit 38% Creatinine 1.0 mg/dL Hemoglobin 12.9 g/dL Na+ 142 mEq/L WBC 7800/mm3 K+ 4.1 mEq/L Cl− 99 mEq/L HCO3 − 24 mEq/L Glucose 102 mg/dL ECG shows atrial fibrillation with a rapid ventricular response. Chest x-ray shows a large left pleural effusion. Which of the following is the most appropriate next step in evaluation? (A) Bone marrow biopsy (B) Pericardiocentesis (C) Pleurodesis (D) Thoracentesis (E) Video-assisted thoracoscopy

**AI_C:** RESTATING THE QUESTION: A 70-year-old woman with a history of hypertension and hyperlipidemia is presenting with worsening shortness of breath over the past month. She is now in respiratory distress with abnormal vitals and pulse oximetry. Her lung exam reveals abnormal percussion on the left side, and her lab results show slightly elevated BUN. The ECG reveals atrial fibrillation with a rapid ventricular response and the chest x-ray demonstrates a large left pleural effusion. The question being asked is what should be the next step in her evaluation. PLANNING: Several assumptions can be made from the patient's presentation. One assumption is that her shortness of breath is related to her pleural effusion. Another assumption is that she has a cardiac origin for her problem, given her atrial fibrillation and elevated BUN. However, the main assumption should be that the pleural effusion is the cause of her symptoms, considering its large size and her acute respiratory distress. Other options related to potential underlying malignancy (bone marrow biopsy, video-assisted thoracoscopy) or cardiac pathology (pericardiocentesis) should be secondary. CLINICAL REASONING: Given the clinical findings, including the dullness to percussion on the left and the large pleural effusion on chest X-ray coinciding with the presence of respiratory distress, pleural effusion seems to be the primary cause of the patient's symptoms. Atrial fibrillation, while concerning, might not be directly related to the primary immediate cause of the symptoms. Rather, it could be a reaction to the underlying condition, or may be contributing to but not solely responsible for her respiratory issues. CONCLUDING: Thus, the most clinically appropriate next step should be the evaluation and management of the pleural effusion. To evaluate the cause of this effusion and to relieve her symptoms, a procedure that directly addresses the effusion would be most suitable. The most appropriate procedure for this would be a thoracentesis, allowing for both immediate relief via the removal of fluid and laboratory evaluation of the fluid to determine its cause. RESPONSE: Therefore, the most appropriate next step in evaluating this 70-year-old woman with respiratory distress and a large left pleural effusion would be to perform a thoracentesis (Option D).

**AI_E:** RESTATING THE QUESTION: This consultation revolves around an elderly female patient with a past significant history of hypertension and hyperlipidemia who is presenting with prominent shortness of breath. The data provided indicates that she is in respiratory distress and has displayed an irregular, accelerated heart rhythm known as atrial fibrillation. Furthermore, on physical examination, there is a projection of dullness to percussion on the left side of her chest which along with her chest x-ray results suggest there is a large pleural effusion occurring. The task is to evaluate and hypothesize the most suitable next step for the patient's care. PLANNING: A large pleural effusion, in this case, can be caused due to several reasons, which include congestive heart failure, renal disease, infection (pneumonia), malignancy, and pulmonary embolism. The laboratory results don't indicate kidney disease or pneumonia, and congestive heart failure is a significant possibility given her cardiovascular risk factors (hypertension, dyslipidemia), atrial fibrillation, and respiratory distress. A diagnosis of malignancy requires more data unless it would be merely speculative. Pulmonary embolism seems less likely given the absence of common symptoms or signs such as a sudden onset, chest pain, calf pain, leg swelling, or cough. CLINICAL REASONING: Given the shortness of breath, tachypnea, tachycardia, reduced oxygen saturation, and chest x-ray findings, a likely assumption is that the patient has a large pleural effusion contributing to her symptoms. Thoracentesis is a procedure that involves inserting a needle into the pleural space between the lungs and the chest wall to remove the excess fluid, which can considerably improve her respiratory distress. Also, this procedure would provide a fluid sample for analysis, which can further identify the aetiology of the effusion (eg, exudate vs. transudate, malignant cells, infection). CONCLUDING: Based on the clinical presentation of respiratory distress, the examination finding of dullness to percussion three-quarters of the way up on the left, concomitant with chest x-ray showing a large pleural effusion, the most logical next step in diagnosis and treatment would be a thoracentesis. This procedure will relieve her symptoms and facilitate further diagnostics. RESPONSE: Thus, the most appropriate next step in evaluation would be (D) Thoracentesis.

**AI_A:** RESTATING THE QUESTION: A 70-year-old woman with a history of hypertension and hyperlipidemia is experiencing worsening shortness of breath and respiratory distress. Her vital signs and lab results indicate irregular pulse, low oxygen saturation, and a large left pleural effusion. Pleural effusion is a buildup of excess fluid between the layers of the pleura, the membranes that line the lungs and inside of the chest wall. Her electrocardiogram presents with atrial fibrillation with a rapid ventricular response - a condition that has an erratic heartbeat that can lead to blood clots, stroke, heart failure and other heart-related complications. Our task then is to identify the most appropriate next step in evaluating her condition from the listed options: bone marrow biopsy, pericardiocentesis, pleurodesis, thoracentesis, or video-assisted thoracoscopy. PLANNING: Given the clinical data, we have to assume that the patient presents with shortness of breath related to the heart or lung pathology, particularly considering the left pleural effusion. However, given her irregularly irregular pulse and low oxygen saturation in the context of left pleural effusion, another assumption could be that the patient has a heart condition which led to the lung problem. The rapid ventricular response may have led to heart failure on the left side, producing the effusion. And, the hypertension could have contributed to this. The next assumption must be clinically prioritized to understand the origin of this effusion and thus, appropriate therapy can be initiated. CLINICAL REASONING: Clinical thought leads to the idea that an intervention aiming to explore the nature of the effusion would be valuable. Thoracentesis involves the withdrawal of fluid from the pleural space and could help determine the cause of the effusion. A pleurodesis, on the other hand, is generally a treatment procedure aimed at causing the pleural surfaces to adhere to each other to prevent reaccumulation of fluid, and thus would not provide the necessary diagnostic information at this point. A bone marrow biopsy would have no clear indication here, as the hematology parameters are within normal limits. Pericardiocentesis involves the removal of fluid from the sac surrounding the heart and might not be appropriate given the location of the effusion (residing around the lungs, not the heart). Finally, video-assisted thoracoscopy is generally reserved for when less invasive procedures fail to give a definitive diagnosis. CONCLUDING: Taking together the results of vital signs, ECG, lab studies, and chest x-ray, and considering the woman's age and medical history, the best solution to discover the origin of her shortness of breath and respiratory distress would be a diagnostic procedure that directly investigates her large left pleural effusion. RESPONSE: Based on a systematic clinical reasoning process, the appropriate next step in evaluation in this case would be (D) Thoracentesis. This procedure will allow for fluid analysis to better understand the etiology of the pleural effusion and guide further management.

**AI_D:** RESTATING THE QUESTION: A 70-year-old woman, with a history of hypertension and hyperlipidemia on treatment with atorvastatin and lisinopril, has reported worsening breathlessness over the past month. She seems to be in respiratory distress at the moment. She is found to have atrial fibrillation (AF) with a rapid ventricular response on an ECG. Her chest X-ray reveals a large left-sided pleural effusion. The patient's blood tests don't indicate any gross abnormalities. We need to decide what the next best step would be to further evaluate her condition. The options are: bone marrow biopsy, pericardiocentesis, pleurodesis, thoracentesis, or video-assisted thoracoscopy. PLANNING: From the given information, it's possible that the cause of the patient's symptoms could be due to her heart disease (AF) not being controlled, leading to heart failure and secondary lung issues. Another explanation could be a different underlying pulmonary disease causing effusion, that is not related to her AF. Alternatively, the pleural effusion could have occurred due to some other systemic disease. Each of these assumptions needs to be confirmed and evaluated for its likelihood. In my opinion, the first step would be to identify the nature of the pleural effusion she has, as it seems to be the direct cause of her acute respiratory symptoms. CLINICAL REASONING: Atrial fibrillation can cause heart failure, which can lead to pleural effusion due to increased venous vascular pressure. This would usually happen bilaterally, though, and she has a left-sided pleural effusion, which makes this less likely to be the primary cause. The pathology behind a pleural effusion resides either in the lung (pneumonia, lung cancer, tuberculosis) or it's related to a systemic disease. Due to the magnitude of her monolateral pleural effusion and her acute respiratory symptoms, it seems pressing to identify the nature of the effusion first. A thoracentesis would take a sample of the pleural fluid and allow us to investigate its cause. CONCLUDING: It seems the first thing to do is assess the nature of her large pleural effusion as this seems to be the cause of her acute symptoms. A thoracentesis is the best way to achieve this, as it would provide a sample of the pleural fluid, which we could test for the presence of infection, malignancy, or other diagnoses. It's less invasive than a thoracoscopy, is more specific than pericardiocentesis and unlike pleurodesis and bone marrow biopsy, it's diagnostic. RESPONSE: Given the circumstances, I suggest that the most appropriate next step in managing this case would be to perform a (D) Thoracentesis, to evaluate the nature of the pleural effusion, as the uncertainty around this seems to be the most likely cause of her acute symptoms. The results from the fluid's analysis would guide us in deciding the subsequent therapeutic approach.

**AI_B:** RESTATING THE QUESTION: The current case represents a 70-year-old woman who is suffering from progressively worsening shortness of breath over the past month. She has an underlying history of hypertension and hyperlipidemia and is undergoing treatment with atorvastatin and lisinopril. Upon examination, she was found to be in respiratory distress with a respiration rate of 28/min and oxygen saturation of 90% on room air, a fever of 36.7°C (98.0°F), and a heart rate of 122/min with irregularly irregular rhythm. The blood pressure recorded was 144/88 mm Hg. An examination of her lungs revealed a dullness to percussion three-quarters of the way up on the left signifying possible pleural effusion. Her lab results returned early and showed the following details. The ECG report indicates atrial fibrillation accompanied by rapid ventricular response and the chest x-ray reveals a large left pleural effusion. Consequently, the current query pertains to which would be the most suitable next step for further investigation, given the options as a bone marrow biopsy, pericardiocentesis, pleurodesis, thoracentesis, or video-assisted thoracoscopy. PLANNING: One of the significant assumptions in this case is the presence of a large left pleural effusion as evidenced by the dullness to percussion found during the physical examination and later confirmed by the chest x-ray. It's imperative we confirm whether the effusion is transudate (due to systemic diseases like congestive heart failure, liver cirrhosis or nephrotic syndrome) or exudate (infections, malignancies, autoimmune diseases). Following that, we can direct our clinical management accordingly. CLINICAL REASONING: Given the presence of a large pleural effusion in the left lung as per the x-ray findings, dullness on percussion, and associated symptoms like shortness of breath and respiratory distress, it is essential to assess the nature of this effusion. While 'Bone marrow biopsy' and 'Pericardiocentesis' would not be directly relevant since no related clinical findings or history suggest a need for them. 'Pleurodesis' is generally performed as a treatment to prevent recurrent pleural effusion or pneumothorax but is not typically used as a diagnostic tool. 'Video-assisted thoracoscopy' is usually reserved for cases wherein a sample for biopsy is needed or when first-line diagnostic options do not provide conclusive results. CONCLUDING: Considering the patient's clinical findings and history, we aim to determine the nature of the pleural effusion, whether it's a transudate, commonly secondary to heart failure, cirrhosis, or nephrotic syndrome, or an exudate, which may suggest malignancy, infection, or autoimmune disease. Thoracentesis, a procedure in which a needle is inserted into the pleural space to remove fluid and relieve symptoms or to obtain a fluid sample for diagnostic purposes, would be the most appropriate next step. RESPONSE: Therefore, in light of the patient's symptoms and our objective to ascertain the type of the detected pleural effusion, 'Thoracentesis' (D) should be the next step in evaluation of this patient.

**Facilitator** Consensus Statement: The case in question involves a 70-year-old woman with a history of hypertension and hyperlipidemia, who is experiencing worsening shortness of breath and respiratory distress. Her vital signs and lab results indicate irregular pulse, low oxygen saturation, and a large left pleural effusion. Her electrocardiogram presents with atrial fibrillation with a rapid ventricular response. Given the clinical data, it is assumed that the patient's shortness of breath is related to heart or lung pathology, particularly considering the left pleural effusion. However, the irregular pulse and low oxygen saturation in the context of left pleural effusion suggest that the patient may have a heart condition which led to the lung problem. The rapid ventricular response may have led to heart failure on the left side, producing the effusion. The hypertension could have contributed to this. The next assumption must be clinically prioritized to understand the origin of this effusion and thus, appropriate therapy can be initiated. Clinical reasoning leads to the idea that an intervention aiming to explore the nature of the effusion would be valuable. Thoracentesis involves the withdrawal of fluid from the pleural space and could help determine the cause of the effusion. Other options such as a bone marrow biopsy, pericardiocentesis, pleurodesis, or video-assisted thoracoscopy are less appropriate given the current clinical context. Taking together the results of vital signs, ECG, lab studies, and chest x-ray, and considering the woman's age and medical history, the best solution to discover the origin of her shortness of breath and respiratory distress would be a diagnostic procedure that directly investigates her large left pleural effusion. Therefore, based on a systematic clinical reasoning process, the appropriate next step in evaluation in this case would be Thoracentesis. This procedure will allow for fluid analysis to better understand the etiology of the pleural effusion and guide further management.
